# Increased COVID-19 infections in women with polycystic ovary syndrome: a population-based study

**DOI:** 10.1530/EJE-20-1163

**Published:** 2021-02-25

**Authors:** Anuradhaa Subramanian, Astha Anand, Nicola J Adderley, Kelvin Okoth, Konstantinos A Toulis, Krishna Gokhale, Christopher Sainsbury, Michael W O’Reilly, Wiebke Arlt, Krishnarajah Nirantharakumar

**Affiliations:** 1 Institute of Applied Health Research, University of Birmingham, Birmingham, UK; 2 Royal College of Surgeons in Ireland (RCSI), University of Medicine and Health Sciences, Dublin, Republic of Ireland; 3 Institute of Metabolism and Systems Research, University of Birmingham, Birmingham, UK; 4 National Institute for Health Research (NIHR), Birmingham Biomedical Research Centre, University Hospitals Birmingham NHS Foundation Trust and University of Birmingham, Birmingham, UK; 5 Midlands Health Data Research UK, Birmingham, UK

## Abstract

**Objective:**

Several recent observational studies have linked metabolic comorbidities to an increased risk from COVID-19. Here we investigated whether women with PCOS are at an increased risk of COVID-19 infection.

**Design:**

Population-based closed cohort study between 31 January 2020 and 22 July 2020 in the setting of a UK primary care database (The Health Improvement Network, THIN).

**Methods:**

The main outcome was the incidence of COVID-19 coded as suspected or confirmed by the primary care provider. We used Cox proportional hazards regression model with stepwise inclusion of explanatory variables (age, BMI, impaired glucose regulation, androgen excess, anovulation, vitamin D deficiency, hypertension, and cardiovascular disease) to provide unadjusted and adjusted hazard risks (HR) of COVID-19 infection among women with PCOS compared to women without PCOS.

**Results:**

We identified 21 292 women with a coded diagnosis of PCO/PCOS and randomly selected 78 310 aged and general practice matched control women. The crude COVID-19 incidence was 18.1 and 11.9 per 1000 person-years among women with and without PCOS, respectively. Age-adjusted Cox regression analysis suggested a 51% higher risk of COVID-19 among women with PCOS compared to women without PCOS (HR: 1.51 (95% CI: 1.27–1.80), *P* < 0.001). After adjusting for age and BMI, HR reduced to 1.36 (1.14–1.63)], *P* = 0.001. In the fully adjusted model, women with PCOS had a 28% increased risk of COVID-19 (aHR: 1.28 (1.05–1.56), *P* = 0.015).

**Conclusion:**

Women with PCOS are at an increased risk of COVID-19 infection and should be specifically encouraged to adhere to infection control measures during the COVID-19 pandemic.

**Significance statement:**

Women with polycystic ovary syndrome (PCOS) have an increased risk of cardio-metabolic disease, which have been identified as a risk factor for COVID-19. To investigate whether the increased metabolic risk in PCOS translates into an increased risk of COVID-19 infection, we carried out a population-based closed cohort study in the UK during its first wave of the SARS-CoV-2 pandemic (January to July 2020), including 21 292 women with PCOS and 78 310 controls matched for sex, age and general practice location. Results revealed a 52% increased risk of COVID-19 infection in women with PCOS, which remained increased at 28% above controls after adjustment for age, BMI, impaired glucose regulation and other explanatory variables.

## Introduction

The novel severe acute respiratory syndrome coronavirus-2 (SARS-CoV-2) reached a pandemic status in March 2020 with a consequent severe impact on international healthcare systems and the global economy (1). The resulting coronavirus disease 2019 (COVID-19) causes mild symptoms in most cases but the incidence of severe illness, respiratory failure and mortality in high-risk groups has led to mandated quarantine measures and economic shutdown across the globe in order to protect capacity within health systems and intensive care units (2, 3). Multiple large observational studies have shown that those with metabolic risk factors such as diabetes, obesity and cardiovascular disease are at higher risk of severe COVID-19 infection (2, 3, 4, 5). Shielding strategies are recommended for older patients and for those with significant comorbidities that place them in a high-risk bracket for severe COVID-19 infection, including being immunocompromised or pregnant or for those with health conditions such as diabetes, heart, liver and lung disease.

Women with polycystic ovary syndrome (PCOS) have recently been highlighted as an overlooked and potentially high-risk population for COVID-19 complications (6). PCOS is a lifelong metabolic condition of women, typically associated in most cases with androgen excess, anovulatory infertility and polycystic ovarian morphology on ultrasound (7, 8, 9). PCOS has an estimated population prevalence between 8–16% of all women, depending on the population studied (10, 11). Women with PCOS are at significantly increased risk of type 2 diabetes mellitus (T2DM) (12, 13, 14), non-alcoholic fatty liver disease (NAFLD) (15) and cardiovascular disease (16). PCOS prevalence is also notably higher in black and South Asian women than in white women (17), the former appears to have a higher risk of severe COVID-19 (18, 19, 20, 21, 22). Whilst younger age and female sex are typically associated with a lower overall risk of severe COVID-19 infection and mortality (2, 4), patients with PCOS may represent a distinct subgroup of women at higher than average risk of adverse COVID-19-related outcomes. It is, therefore, imperative to determine whether PCOS is linked to COVID-19 susceptibility.

We hypothesized that women with PCOS are at a higher risk of development of COVID-19 compared to an age-matched control population. Our aim was to examine the incident risk of reported suspected/confirmed COVID-19 in women with PCOS in the UK utilizing a large primary care database, in comparison to matched population controls.

## Methods

### Study design and data source

A population-based retrospective closed cohort study to determine the incident risk of COVID-19 infection in women with PCOS in comparison to women without PCOS was conducted in The Health Improvement Network (THIN) database. THIN is an anonymized longitudinal primary care electronic medical records database from 365 active general practices in the UK. The records include patient demographics data, symptoms, diagnoses, drug prescriptions, physical measurements and laboratory test results. Symptoms and diagnoses are recorded using Read codes, a hierarchical coding system (23). We have previously conducted studies examining long-term outcomes of women with PCOS using the THIN database (15, 24).

### Study population

Women aged 18 and above were included if they had a minimum registration period of 1 year with an eligible general practice to maximize completeness of baseline records. Patient age at study entry (31 January 2020) was not restricted to reproductive age considering the lifelong metabolic disturbances associated with this condition. Women with a coded diagnosis of PCOS or polycystic ovaries (PCO) before study entry were included in the PCOS cohort. For the purpose of this study, women with a coded diagnosis of PCO were considered as women with PCOS as previous studies have highlighted that these codes have been interchangeably recorded in the primary care electronic medical records in the UK (25). Read codes for both PCOS and PCO are listed in Supplementary Table 1A (see section on supplementary materials given at the end of this article). Women who are pregnant at study entry were excluded from cohort selection as they are more likely to be tested for COVID-19, due to systematic screening during admission for delivery (26), which could affect the primary outcome.

**Table 1:** Baseline characteristics of women with PCOS and age-matched controls.

	Exposed (PCO/PCOS)	Unexposed	*P*-value
*n*	21 292	78 310	
Age			
Mean ± s.d.	39.3 ± 11.1	39.5 ± 11.3	0.030^¥^
Median (IQR)	38.5 (30.5–46.5)	38.5 (30.5–47.5)	
Categories, years			0.009^§^
18–30	4697 (22.1)	17 403 (22.2)	
30–40	7170 (33.7)	25 591 (32.7)	
40–50	5658 (26.6)	20 736 (26.5)	
50–60	2951 (13.9)	11 358 (14.5)	
>60	816 (3.8)	3222 (4.1)	
BMI			
Mean ± s.d.	31.0 ± 8.4	27.1 ± 6.7	<0.001^¥^
Median (IQR)	29.7 (24.4–36.2)	25.50 (22.3–30.4)	
Categories			<0.001^§^
Normal/underweight (<25 Kg/m^2^)	5530 (26.0)	31 671 (40.4)	
Overweight (25–30 Kg/m^2^)	4494 (21.1)	18 112 (23.1)	
Obese (>30Kg/m^2^)	9538 (44.8)	17 837 (22.8)	
Missing	1730 (8.1)	10 690 (13.7)	
Androgen excess*	4849 (22.8)	1399 (1.8)	<0.001^§^
Testosterone ≥ 2.0 nmol/L	2552 (12.0)	665 (0.9)	<0.001^§^
Hirsutism	2838 (13.3)	786 (1.0)	<0.001^§^
Anovulation	5867 (27.6)	5770 (7.4)	<0.001^§^
IGR categories			<0.001^§^
Absence of IGR	18 767 (88.14)	74 590 (95.25)	
Pre-diabetes	873 (4.10)	1673 (2.14)	
Diabetes	1652 (7.76)	2047 (2.61)	
Vitamin D deficiency	627 (3.0)	1398 (1.8)	<0.001^§^
Hypertension	2023 (9.5)	4404 (5.6)	<0.001^§^
Composite CVD^¬^	45 (1.6)	984 (1.3)	<0.001^§^
Ischaemic heart disease	175 (0.8)	484 (0.6)	0.001^§^
Stroke/TIA	128 (0.6)	381 (0.5)	0.038^§^
Heart failure	51 (0.2)	139 (0.2)	0.066^§^
Peripheral vascular disease	31 (0.2)	91 (0.1)	0.277^§^

*Hirsutism/testosterone ≥ 2.0 nmol/L; ^¬^Ischaemic heart disease/stroke/TIA/heart failure/peripheral vascular disease; ^¥^*P*-value obtained from *t*-test comparing means of the variable between the two groups; ^§^*P*-value obtained from chi-square test comparing the percentage of women in each category between the two groups.

IGR, impaired glucose regulation; TIA,transient ischaemic attack.

For every woman with PCOS, we randomly selected four women without a diagnostic code for PCOS/PCO and matched for age (±1 year) and general practice location.

### Outcome and follow-up

The primary outcome was a composite of suspected or confirmed diagnosis of COVID-19 in primary care; Read codes are listed in Supplementary Table 1B. According to NHS Guidance and Standard Operating Procedures for Primary Care, and UK Faculty of Clinical Informatics guidelines, confirmed COVID-19 codes represent a positive RT-PCR test, while a suspected COVID-19 code represents a symptomatic presentation of COVID-19 and/or contact history with a confirmed patient (27). Considering the wide unavailability of RT-PCR tests outside of the hospital setting until relatively later in the initial wave of COVID-19 pandemic in the UK, most cases of COVID-19 in primary care are coded as ‘suspected’. All women included in the study were followed up from 30 January 2020 (index date) until patient exit date: a patient was considered to exit the study at the earliest of the suspected/confirmed COVID-19 infection documentation date or the patient being lost to follow-up (i.e. patient deregistration from the practice or patient death) or study end date (22 July 2020, last date of data provided by Cegedim, the THIN data provider).

### Explanatory variables

We considered PCOS features that overlap with COVID-19 infection risk as explanatory variables, which included age, BMI, impaired glucose regulation, androgen excess, anovulation (lack of regular ovulation or symptomatic sequelae of anovulation) , vitamin D deficiency at baseline and concurrent diagnosis of hypertension and cardiovascular diseases, informed by previously identified COVID-19 risk factors (6). Age was categorized into 10-year age bands: 18–30, 30–40, 40–50, 50–60 and 60+ years. BMI was considered as a continuous variable. Multiple imputation using chained equations and predictive mean matching were performed to replace missing BMI values. Impaired glucose regulation was categorized as either (i) absence of diabetes, (ii) pre-diabetes or (iii) diabetes (identified by either Read code records or the HbA1c measurement at baseline (42 to 47 mmol/mol (6.0–6.4%) for pre-diabetes and ≥48 mmol/mol (≥6.5%) for diabetes). Androgen excess was defined as the latest serum testosterone measurement ≥2.0 nmol/L at baseline and/or the presence of hirsutism. Vitamin D deficiency was identified by Read codes. Cardiovascular disease was defined as a composite of ischaemic heart disease, heart failure, stroke, transient ischaemic attack and peripheral vascular disease.

### Statistical analysis

Description of baseline variables is provided using appropriate summary statistics stratified by PCOS vs non-PCOS mean with standard deviation (s.d.) and median with interquartile range (IQR) were provided for continuous variables as appropriate. Frequency and percentage were provided for categorical variables. *T*-test and chi-square test were used to test for statistically significant differences in the baseline variables between PCOS and non-PCOS.

A Cox proportional hazards regression model was used to provide unadjusted and adjusted hazard ratios (HRs) of the primary outcome among women with PCOS compared to women without PCOS after stepwise inclusion of the explanatory variables in the Cox model, culminating with a fully adjusted model.

We performed two sets of sensitivity analyses to assess the robustness of our findings. First, we restricted the exposed cohort to patients with a coded diagnosis of PCOS only (instead of PCOS/PCO) and performed the Cox regression analysis along with their matched controls. Secondly, we restricted the analyses to patients of reproductive age (18–50) at study entry and through the study period.

All analyses were performed in Stata IC version 15. Two-sided *P* values were obtained for all tests, and a *P* value <0.05 was considered as statistically significant. Selection of Read code lists was performed using methods used in our previous publications (28).

### Ethics

The THIN data collection scheme and research carried out using THIN data were approved by the NHS South–East Multicentre Research Ethics Committee in 2003. Under the terms of the approval, studies must undergo independent scientific review. Approval for this study was obtained from the THIN Scientific Review Committee in September 2020 (SRC protocol reference 20-010).

## Results

### Characteristics of the cohort of women with PCOS and their age-matched controls

As of 31 January 2020, 326 practices out of 365 practices qualified for inclusion with 1 012 944 registered women aged 18 and above. We identified 8103 women with a coded diagnosis of PCOS and 13 189 additionally with a coded diagnosis of PCO. From a pool of 969 162 women eligible to be in the control population, a total of 78 310 women were randomly selected as controls, matched for age and GP surgery location.

The mean (s.d.) age at study entry of the women with and without PCOS was 39.3 (11.1) and 39.5 (11.3), respectively (Table 1). Among the women with PCOS, the mean (s.d.) age at diagnosis of PCOS and mean (s.d.) duration after the diagnosis of PCOS at study entry were 27.0 (7.0) and 12.4 (8.9) years, respectively.

As anticipated, there were significantly higher levels of all characteristic features of PCOS among the women with PCOS than in the matched controls (Table 1). Out of the women with a record of BMI at baseline (91.9% and 86.3% among women with and without PCOS), women with PCOS had significantly higher BMI compared to women without PCOS (mean (s.d.): 31.0 (8.4) vs 27.1 (6.7), *P* < 0.001). Androgen excess, defined as a coded diagnosis of hirsutism and/or the latest recorded serum testosterone measurement ≥2.0 nmol/L prior to study entry, was recorded between 22.8% and 1.8% of women with and without PCOS, respectively (*P* < 0.001). A coded diagnosis of anovulation at baseline was recorded between 27.6% and 7.4% of the women with and without PCOS, respectively (*P* < 0.001). At baseline, approximately 7.8% and 4.1% of the women with PCOS had diabetes and pre-diabetes, respectively, while only 2.6% and 2.1% of the control women had records of these conditions (*P* < 0.001). Women with PCOS were more likely to be vitamin D deficient (3.0% vs 1.8%, *P* < 0.001), hypertensive (9.5% vs 5.6%, *P* < 0.001) or have cardiovascular disease at baseline (1.6% vs 1.3%, *P* < 0.001).

### Risk of COVID-19 among women with PCOS compared to their age-matched controls, after adjustment for PCOS features

**Figure 1:**
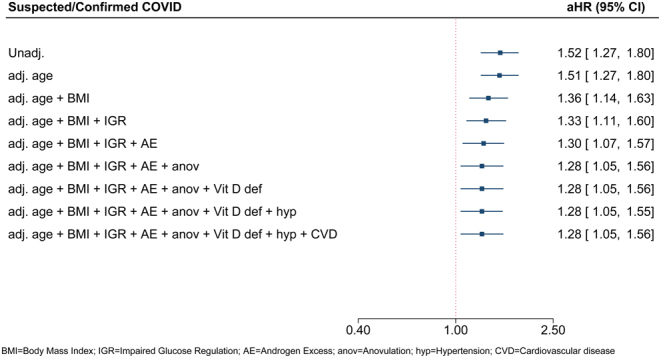
Risk of confirmed/suspected COVID-19 among women with PCOS after serial adjustment for PCOS features. A full colour version of this figure is available at https://doi.org/10.1530/EJE-20-1163.

Among the women with and without PCOS, 0.9% (*n* = 180) and 0.6% (*n* = 438), respectively, had a record of suspected/confirmed COVID-19 in their primary care records during a cumulative follow-up of 9967 and 36 727 person-years, respectively (Table 2). Confirmed COVID-19 codes were only present in 0.1% (*n* = 14) and 0.1% (*n* = 70) of women with and without PCOS, respectively. This provided a crude COVID-19 incidence rate of 18.1 and 11.9 per 1000 person-years among the women with and without PCOS, respectively. An age-adjusted Cox regression analysis suggested a 51% higher risk of suspected/confirmed COVID-19 among women with PCOS compared to women without PCOS (1.51 (95% CI: 1.27–1.80), *P* < 0.001) (Fig. 1).

**Table 2:** Risk of suspected/confirmed COVID-19 among women with PCOS compared to women without PCOS.

	Exposed	Unexposed	HR (95% CI)		*P*-value	
			Unadjusted	Adjusted*	Unadjusted	Adjusted*
Primary analysis, *n*	21 292	78 310	1.52 (1.27–1.80)	1.28 (1.05–1.56)	<0.001	0.015
Outcome events, *n* (%)	180 (0.85)	438 (0.56)				
Person-years	9967	36 727				
CIR/1000 PY	18.06	11.93				
Sensitivity analysis						
Restriction of exposure to PCOS codes only,* n*	8103	29 711	1.59 (1.20–2.10)	1.38 (0.99–1.92)	0.001	0.056
Outcome events, *n* (%)	69 (0.85)	160 (0.54)				
Person-years	3788	13 926				
CIR/1000 PY	18.21	11.49				
Restriction of cohort to women of reproductive age (18–50), *n*	17 525	63 775	1.56 (1.29–2.88)	1.30 (1.05–1.62)	<0.001	0.018
Outcome events, *n* (%)	152 (0.87)	353 (0.55)				
Person-years	8180	29 546				
CIR/1000 PY	18.58	11.95				

*Adjustment for age category, BMI, impaired glucose regulation, androgen excess, anovulation, vitamin D deficiency, hypertension and cardiovascular diseases.

CIR, crude incidence rate.

After adjusting for BMI and age, the hazard ratio reduced to 1.36 (95% CI: 1.14–1.63), *P* = 0.001 (Fig. 1). When additionally adjusting for impaired glucose regulation, the hazard ratio was marginally further reduced to 1.33 (95% CI: 1.11–1.60), *P* = 0.002. Following this, in a series of further stepwise adjustments for androgen excess and anovulation, the hazard ratios were reduced to 1.30 (95% CI: 1.07–1.57), *P* = 0.008 and 1.28 (95% CI: 1.05–1.56), *P* = 0.014 *P* = 0.018, respectively. Additional adjustment for vitamin D deficiency, hypertension and cardiovascular disease made no difference to the effect estimate. In the fully adjusted model, women with PCOS had a 28% increased risk of suspected/confirmed COVID-19 compared to women without PCOS (aHR: 1.28 (95% CI: 1.05–1.56), *P* = 0.015).

When restricting the exposure ascertainment to codes specific to PCOS only, that is excluding PCO codes, a 37% increased risk of suspected/confirmed COVID-19 was observed among women with PCOS (*n* = 8103) compared to their matched controls (*n* = 29 711); although, the results did not reach statistical significance (aHR: 1.38 (95% CI: 0.99–1.92), *P* = 0.056).

In the sensitivity analysis restricting to reproductive-aged women, the results suggest that women with PCOS between the age of 18 and 50 years (*n* = 17 525) have a 30% increased risk of suspected/confirmed COVID-19 compared to women without PCOS matched for age and general practice (*n* = 63 775) (aHR: 1.30 (95% CI: 1.05–1.62), *P* = 0.018) (Table 2).

### Risk factors for COVID-19 among all women

In the fully adjusted model, there was lower risk of reported suspected/confirmed COVID-19 among women aged ≥60 years compared to women aged 18-30 (aHR: 0.41 (95% CI: 0.23–0.74), *P* = 0.001) and 2% higher risk with every unit (kg/m^2^) increase in BMI (aHR: 1.02 (95% CI: 1.01–1.03), *P* < 0.003) (Table 3). Furthermore, there was a higher risk of suspected/confirmed COVID among women who had vitamin D deficiency (aHR: 1.61 (95% CI: 1.05–2.47), *P* = 0.029) or cardiovascular disease (1.88 (95% CI: 1.12–3.17), *P* = 0.017) at baseline. Risk was also higher in the presence of pre-diabetes and diabetes but this did not reach statistical significance (aHR: 1.31 (95% CI: 0.86–2.00), *P* = 0.215 and 1.36 (95% CI: 0.96–1.93), *P* = 0.085, respectively).

**Table 3:** Risk factors for confirmed/suspected COVID-19 from the fully adjusted model.

Risk factors	Adjusted hazard ratio	*P*-values
PCOS	1.28 (1.05–1.56)	0.015
Age category, years		
18–30	RS	RS
30–40	0.89 (0.71–1.06)	0.286
40–50	1.03 (0.82–1.29)	0.785
50–60	0.89 (0.68–1.18)	0.428
≥60	0.41 (0.23–0.74)	0.003
BMI	1.02 (1.01–1.03)	<0.001
Androgen excess	1.11 (0.83–1.50)	0.478
Anovulation	1.06 (0.84–1.35)	0.594
Impaired glucose regulation		
Absence of IGR	RS	RS
Pre-diabetes	1.31 (0.86–2.00)	0.215
Diabetes	1.36 (0.96–1.93)	0.085
Vitamin D deficiency	1.61 (1.05–2.47)	0.029
Hypertension	1.19 (0.88–1.62)	0.258
Cardiovascular disease	1.88 (1.12–3.17)	0.017

RS, reference standard.

## Discussion

In this retrospective cohort study spanning the first wave period of the COVID-19 pandemic in the UK, we found that a diagnosis of PCOS confers a 51% increased risk of development of confirmed or suspected COVID-19 infection compared to the background age-matched female population. A higher observed susceptibility to COVID-19 infection (26%) in the PCOS cohort persisted even after adjustment for individual cardio-metabolic risk factors known to cluster within PCOS, which have recently been directly linked to increased COVID-19 susceptibility including obesity, impaired glucose regulation and androgen excess (2, 6, 29). These data support an independent relationship between a diagnosis of PCOS and risk of COVID-19 infection; however, the precise pathophysiological mechanisms underpinning this association are not clear.

PCOS is a lifelong condition associated with severe health consequences in women, including a significantly increased risk of T2DM, NAFLD and cardiovascular disease (15, 16). To our knowledge, this is the first publication since the pandemic outbreak that has demonstrated an increased susceptibility to COVID-19 infection in women with PCOS. Given the high prevalence of PCOS in the population, these findings need to be considered when designing public health policy and advice as our understanding of COVID-19 evolves. Before the onset of the COVID-19 pandemic, women with PCOS had low rates of satisfaction with access to and provision of healthcare services in relation to their condition (30). Women with PCOS consistently report fragmented care, delayed diagnosis and a perception of poor clinician understanding of their condition as major factors contributing to this dissatisfaction (31). Women suffering from this condition may fear, with some degree of justification, that an enhanced risk of COVID-19 infection will further compromise timely access to healthcare and serve to increase the sense of disenfranchisement, currently experienced by many patients. The pandemic has already dramatically altered our current healthcare delivery models, and although the increased roll-out of virtual consultations and methods of delivering remote healthcare have been commendable, for many patients with PCOS these will not be an appropriate substitute for the traditional clinician–patient live consultation. The risk of mental health problems including low self-esteem, anxiety and depression is significantly higher in women with PCOS than the background female population, and advice on strict adherence to social distancing needs to be tempered by the associated risk of exacerbating these underlying problems.

PCOS is a pro-inflammatory state, and it has been hypothesized that inflammation may underpin many of the cardio-metabolic abnormalities in this disorder (32). Increased circulating levels of pro-inflammatory mediators, including highly sensitive C-reactive protein (hsCRP), tumour necrosis factor (TNF)-alpha, procalcitonin and interleukin-18 (IL-18), have been reported in women with PCOS (33, 34), and although more pronounced in the context of obesity, these associations persist even after correction for total fat mass. Pro-inflammatory cytokines are implicated in adipose tissue dysfunction and inflammation (35) and have been implicated in the pathophysiology of insulin resistance and diabetes (36). Severe COVID-19 infection, with associated respiratory failure requiring oxygen therapy or admission to intensive care for intubation and ventilation, has also been linked with an exaggerated systemic inflammatory response, which can trigger catastrophic acute respiratory distress syndrome (ARDS) with associated multi-organ failure and high mortality. It is conceivable that women with PCOS, who have been demonstrated to have low-grade inflammation beyond that observed in simple obesity , are potentially at increased risk of severe COVID-19 infection because of this underlying pro-inflammatory predisposition (37, 38).

The link between COVID-19 infection and androgens merits further discussion. Androgen excess is a cardinal feature of PCOS and identified as a primary driver of increased risk of T2DM and NAFLD in affected women (15, 39). Significant gender differences have been observed in COVID-19 outcomes with a higher likelihood of hospitalization and death in men reported in multiple studies (37). Intriguingly, androgen deprivation therapy in men treated for prostate cancer was associated with a significantly reduced risk of SARS-COV-2 infection compared to those treated with alternative disease regimens in a recent study (38); a preliminary report from Spain has linked more severe infection with androgenic alopecia in male patients (40). Conversely, a limited number of small studies have also linked low serum testosterone at baseline in hospitalized men to an increased risk of ICU admission and death (41, 42); indeed, it is intriguing that the metabolic complications associate with male hypogonadism mirror those of women with androgen excess (43). Early *in vitro* studies suggest that the transmembrane serine protease 2 (TMPRSS2), which is highly regulated by androgens, is a critical enzyme mediating the entry of the SARS-CoV-2 into cells (44). It is reasonable to speculate that women with PCOS and androgen excess are at increased susceptibility of infection through this mechanism. Whilst androgen excess was not identified as a major contributor to COVID-19 susceptibility in our PCOS cohort, it is likely to be the subject of increased clinical research interest in the months and years ahead. In addition, it is a limitation of our study that we had to base the diagnosis of androgen excess on surrogate parameters, hirsutism and serum testosterone concentrations. Testosterone has not been systematically measured in our PCOS cohort with no data available on 11-oxygenated androgens, the predominant circulating androgens in PCOS (45). Androgens are important modulators of immune function (46), and very recent observations have highlighted that peripheral blood mononuclear cells preferentially activate 11-oxygenated androgens and that natural killer cells, the prime innate defence against viral infection, represent the major site of this intracrine androgen activation (47).

Strengths of our study included a large sample size from a dataset generalizable to the UK population and the study period covers the majority of the COVID-19 pandemic duration in the UK to date. The proportion of missing information was low, and we adjusted for a range of potential confounders, in a stepwise series of regression models; however, there are several important limitations. The data quality is dependent on accurate coding by general practitioners and primary care administrative staff; there is a possibility of miscoding of the PCOS/PCO diagnosis, and recording of suspected or confirmed COVID-19 may be incomplete.

A considerable limitation in this study was the restriction of PCOS ascertainment using clinical codes recorded by a general practitioner. Endocrinological evaluation is more likely to be performed by a specialist in secondary care, while the GP may limit to the coding of a confirmed diagnosis. Therefore, the prevalence of PCOS observed in primary care setting is usually under recorded rather than over diagnosed. Importantly, it was not possible to explore or adjust for the effect of patient ethnicity or socioeconomic status, as this data were unavailable. While we have adjusted for a number of important confounders, there remains a possibility of unmeasured confounding. Also, confounders such as androgen excess and impaired glucose regulation were restricted to clinical coding and available measurements such as serum testosterone and HbA1c, which may not have captured the complete picture of metabolic disturbances. Finally, during the first wave of COVID-19 in the UK there was no widespread testing in primary care with a COVID-19 test generally only being performed if a patient was admitted to hospital; we have, therefore, presented a combination of confirmed and clinically suspected COVID-19 infections.

In conclusion, our study shows that women with PCOS are at an increased risk of COVID-19 infection, and except for obesity the adjustment for potentially confounding factors did not mitigate this risk, pointing at inherent PCOS-specific factors. Future studies should explore the potentially critical role of androgens in conveying this risk and assess in more detail the contribution of ethnicity and socio-economic deprivation. Based on our results, women with PCOS should be specifically encouraged to adhere to the recommended infection control measures for the duration of the COVID-19 pandemic.

## Supplementary Material

Supplementary Table 1A: ReadCodes used for the ascertainment of exposure status (PCO/PCOS)

## Declaration of interest

Wiebke Arlt is on the editorial board of EJE. Wiebke Arlt was not involved in the review or editorial process for this paper on which he/she is listed as an author.

## Funding

This study was funded by Health Data Research UK and supported by the Wellcome Trust (Investigator Grant WT209492/Z/17/Z, to W A) and the Health Research Board (HRB; Emerging Clinician Scientist Award ECSA-2020-001, to M W O’R). WA receives support from the NIHR Birmingham Biomedical Research Centre at the University Hospitals Birmingham NHS Foundation Trust and the University of Birmingham (Grant Reference Number BRC-1215-20009). The views expressed are those of the authors and not necessarily those of the NIHR UK or the Department of Health and Social Care UK.
