# Supplementary material for: Increased COVID-19 infections in women with polycystic ovary syndrome: a population-based study
Source: Eur J Endocrinol. 2021 May 1;184(5):637–45. doi: 10.1530/EJE-20-1163 (PMC8052516; doi:10.1530/EJE-20-1163)
Supplement: EJE-20-1163-supplementary_table_1 — Supplementary Table 1A: ReadCodes used for the ascertainment of exposure status (PCO/PCOS) [file eje-20-1163-supplementary_table_1.pdf]

Supplementary Table 1A: ReadCodes used for the ascertainment of exposure status (PCO/PCOS)

Code	Description
C165.00	Polycystic ovarian syndrome
C164.00	Polycystic ovaries
C164.12	Stein - Leventhal syndrome
C164.13	Multicystic ovaries

Supplementary Table 1B: ReadCodes used for the ascertainment of outcome (confirmed/suspected COVID)

Code	Description
G558500	Cardiomyopathy due SARS-CoV-2
A795200	COVID-19 confirmed by laboratory test
G520800	Myocarditis due to SARS-CoV-2
H051100	URTI due to SARS-CoV-2
A076400	Gastroenteritis due to SARS-CoV-2
F529.00	Otitis media due to SARS-CoV-2
F289.00	Encephalopathy due to SARS-CoV-2
A795300	COVID-19 confirmed using clinical diagnostic criteria
H204.00	Pneumonia due to SARS-CoV-2
43hF.00	Detection of SARS-CoV-2 by PCR
4J3R100	2019-nCoV (novel coronavirus) detected
9N31200	Telephone consultation for suspected 2019-nCoV (novel coronavirus)
A795100	Disease caused by 2019-nCoV (novel coronavirus)
1JX1.00	Suspected disease caused by 2019-nCoV (novel coronavirus)
1JX..00	Suspected coronavirus infection
43dt400	Has immunity to SARS-CoV-2
43dtA00	SARS-CoV-2 IgG detected
43dtG00	SARS-CoV-2 IgM detected
